# Estimating helmet wearing rates via a scalable, low-cost algorithm: a novel integration of deep learning and google street view

**DOI:** 10.1186/s12889-024-19118-0

**Published:** 2024-06-20

**Authors:** Qingfeng Li, Xianglong Wang, Abdulgafoor M. Bachani

**Affiliations:** 1 Johns Hopkins International Injury Research Unit, Health Systems Program, Department of International Health, Johns Hopkins Bloomberg School of Public Health, Baltimore, MD USA; 2 Johns Hopkins International Injury Research Unit, Health Systems Program, Department of International Health, Johns Hopkins Bloomberg School of Public Health, 615 North Wolfe Street, E-8136, Baltimore, MD 21205 USA

**Keywords:** Helmet, Motorcyclists, Deep learning, Google Street View, Low-cost and scalable algorithm

## Abstract

**Introduction:**

Wearing a helmet reduces the risk of head injuries substantially in the event of a motorcycle crash. Countries around the world are committed to promoting helmet use, but the progress has been slow and uneven. There is an urgent need for large-scale data collection for situation assessment and intervention evaluation.

**Methods:**

This study proposes a scalable, low-cost algorithm to estimate helmet-wearing rates. Applying the state-of-the-art deep learning technique for object detection to images acquired from Google Street View, the algorithm has the potential to provide accurate estimates at the global level.

**Results:**

Trained on a sample of 3995 images, the algorithm achieved high accuracy. The out-of-sample prediction results for all three object classes (helmets, drivers, and passengers) reveal a precision of 0.927, a recall value of 0.922, and a mean average precision at 50 (mAP50) of 0.956.

**Discussion:**

The remarkable model performance suggests the algorithm’s capacity to generate accurate estimates of helmet-wearing rates from an image source with global coverage. The significant enhancement in the availability of helmet usage data resulting from this approach could bolster progress tracking and facilitate evidence-based policymaking for helmet wearing globally.

## Introduction

According to the World Health Organization (WHO), road traffic crashes cause about 1.3 million deaths and 50 million injuries annually [1]. Over the past two decades, the level and rate of road traffic crashes have remained relatively steady globally and even increased in some low- and middle-income countries. More than half of all road traffic deaths are among vulnerable road users: pedestrians, cyclists, and motorcyclists [1]. 

Motorcyclists constitute a significant proportion of road traffic injuries and deaths, ranging from 12% in high-income countries to 26% in middle-income countries [2]. Head injuries are the predominant cause of severe injuries and deaths among this type of road user. Compared to other motor vehicles, with which motorcyclists often share the traffic space, the lack of protection from steel shells makes them more susceptible to traumatic brain injuries. Wearing a helmet reduces the risk of death by 42% and the risk of severe injury by 69%.^1^ That proven effectiveness has motivated 167 countries to enforce mandatory helmet laws for motorcyclists [1]. However, the enforcement of helmet-wearing laws is generally suboptimal for various reasons.

A significant gap is the dearth of comprehensive data on helmet-wearing. Large-scale, up-to-date data, whether at the city or national levels, are scarce, posing considerable challenges in assessing the current situation and evaluating the efficacy of helmet-wearing promotion efforts. That has been an important barrier to evidence-based policymaking and monitoring.

Traditionally, observational methods have been employed to gather information on helmet-wearing. However, those methods are costly and difficult to scale, especially considering large and diverse populations. Large-scale data collection can be technically challenging and resource-intensive. Furthermore, observational methods often involve subjective assessment, which can be prone to errors and bias. Consequently, there is a crucial need for low-cost, scalable approaches to collect standardized data on helmet-wearing.

This study aims to address this need through a deep learning-based algorithm for estimating helmet-wearing among motorcyclists. Deep learning has emerged as a powerful technique to deliver impressive performance across diverse tasks in various fields in recent years. Compared to traditional approaches that rely on human labor, deep learning-based methods are renowned for their efficiency and cost-effectiveness. Deep learning has demonstrated significant superiority over traditional methods in object detection due to its capability to learn complex patterns, particularly in computer vision tasks [3]. Previous applications of deep learning algorithms achieved high performance in detecting helmets in images and videos [4–6]. 

Our proposed methodology leverages the benefits of low-cost data acquisition, made possible by utilizing Google Street View APIs. Compared to previous studies that utilize deep learning for helmet wearing estimation, the proposed algorithm’s major advancement lies in its integration of Google Street View as the data source. The dependence on primary data collection in prior studies severely restricts the applicability of their algorithms [7]. The ubiquity and easy accessibility of Google Street View facilitates our approach to be a cost-effective solution with the potential to enhance the availability and quality of helmet-wearing data worldwide substantially. By leveraging these methodologies, our proposed algorithm offers a reliable, low-cost solution for estimating helmet-wearing that can be easily implemented in a multitude of settings.

## Methods

The proposed solution is comprised of two primary modules that work sequentially to obtain and process images.

- Image Acquisition.

**Fig. 1:**
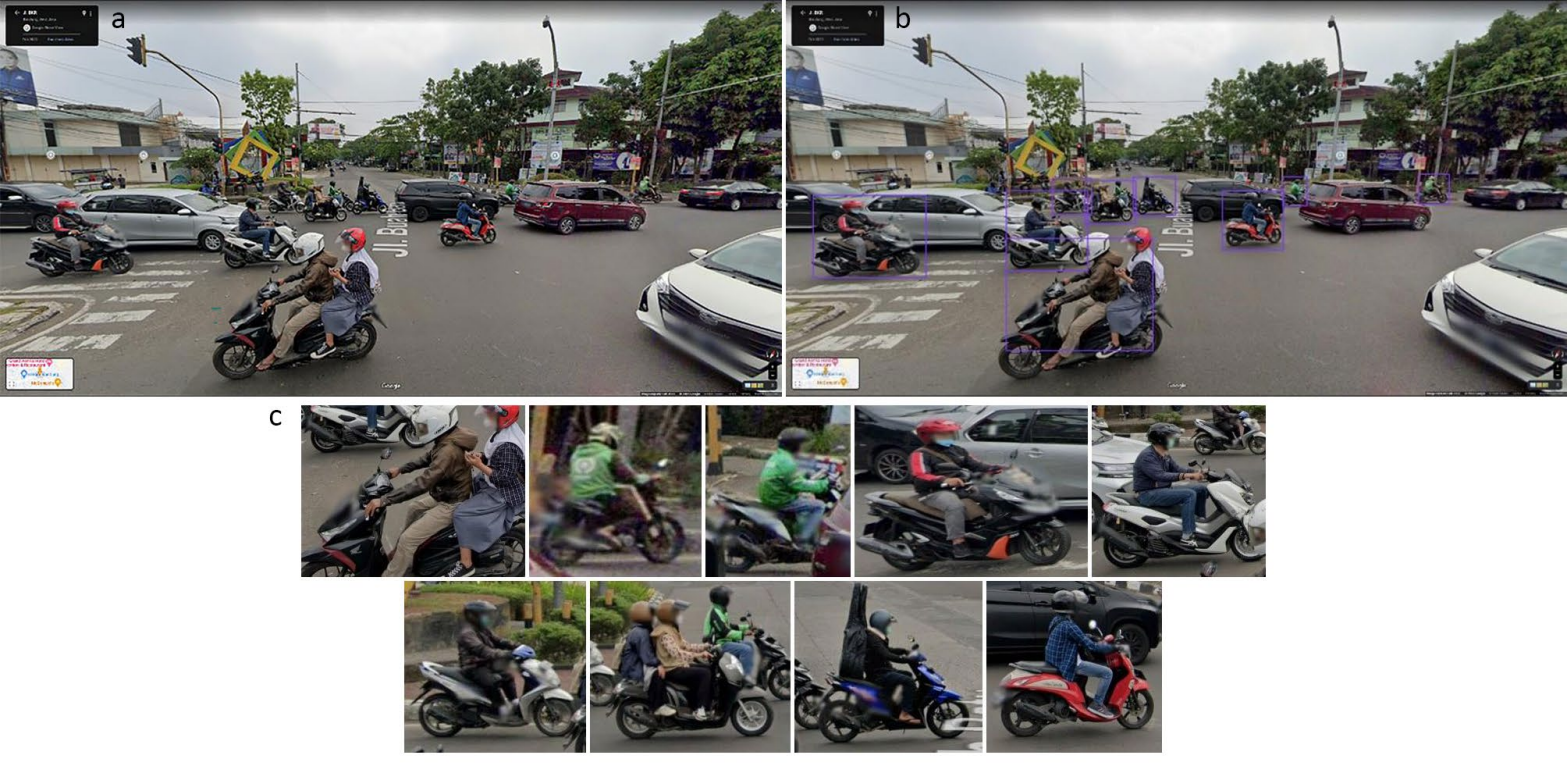
(**a**): Google Street View (**b**): detecting motorcycles using YOLO (**c**): cropped motorcycles Extracting and pre-processing images from google street view

The first module acquires images from Street View for specified locations or regions (Fig. 1). Street View is a feature of Google Maps that provides a 360-degree panoramic view of the surrounding environment, inclusive of vehicles and road users present on roads. Since introducing this feature in 2007, Google has been steadily augmenting its coverage. As of 2022, Street View is accessible for more than 10 million miles of roads in excess of 100 countries and territories around the world [8]. Google also offers a dedicated API for downloading Street View images.

For training and validating the proposed algorithm, we selected Bandung, Indonesia, as the study site due to its significant usage of motorcycles and the high burden of deaths among motorcyclists [9]. About 34% of road safety deaths are motorcyclists in the South-East Asian and Western Pacific Regions [2]. Utilizing the city’s shapefile, we randomly selected intersections across the city and subsequently captured images around the selected locations.

Prior to the training of the deep learning algorithm, images undergo pre-processing. The first phase of pre-processing entails the removal of images devoid of motorcycles. YOLO (You Only Look Once, version 5) was selected as the detection algorithm for this task [10]. YOLO is capable of detecting multiple objects in a single pass through the network and delivering real-time results. Since its 2016 inception, YOLO has undergone considerable refinement in terms of performance and speed. Renowned for its speed and accuracy, YOLO is extensively utilized within the computer vision research community and industry [11–13]. Trained on COCO, one of the most popular object detection datasets, the default specification of YOLO can detect about 80 different categories. Another critical feature of YOLO is that it can be trained on a customized dataset to detect user-defined object classes.

The subsequent phase of image pre-processing involves cropping motorcycles from the images. Formally known as image segmentation or background subtraction, this step potentially improves model performance by reducing input complexity and focusing on objects of interest. Consequently, during training, the algorithm is less affected by noises in the images and more able to concentrate on learning relevant features [14]. Similar pre-processing has been applied and achieved significant model improvement [15–17].

- Object Detection

**Fig. 2:**
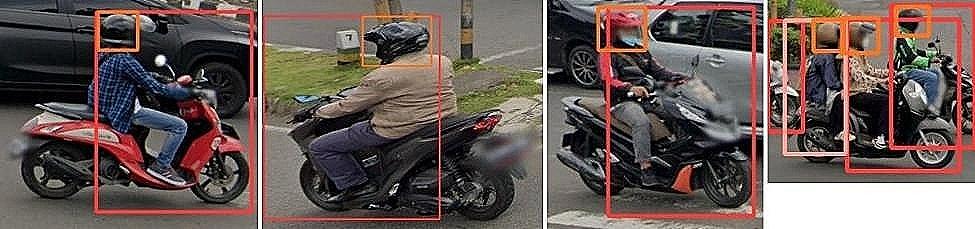
Labeled images with manually drawn bounding boxes around object classes

The second module detects objects of interest from the images, namely helmets, motorcycle drivers, or passengers. The default specification of YOLO is incapable of identifying those object classes. To train YOLO to detect those object categories, we created a custom dataset where those objects are labeled (Fig. 2). The labeling was done manually by drawing bounding boxes around three object categories of interest: helmet, front wheel plus driver, and rear wheel plus passenger. Wheels are included in defining drivers and passengers in order to better differentiate them from other road users, such as pedestrians. There are few motorcycles with more than two riders; therefore, this instance is not accounted for that possibility in object definitions.

For the task of image annotation, we initially employed Amazon Mechanical Turk (AMT), a platform widely recognized and utilized in both industry and academia for various annotation tasks [18, 19]. We required that the workers hold a Masters qualification, a credential granted by Amazon for achieving top-tier performance metrics. These workers have consistently demonstrated high accuracy across a broad spectrum of annotation tasks. Each image was annotated independently by two different AMT workers. Our evaluation revealed an overall inter-rater reliability (IRR) of 0.85. The IRR for the helmet class was nearly perfect, standing at 0.98. Such levels of IRR are considered excellent or almost perfect in the literature [20, 21]. The results demonstrate that it is a simple and straightforward task for humans to identify objects of interest in the images.

However, we noticed an issue with the exact placement of the bounding boxes by AMT workers. The bounding boxes did not always tightly enclose the objects of interest, particularly when the image was captured from a side angle. This characteristic is crucial for our algorithm, given the relatively low resolution of our images. Given the challenge for the AMT workers to rectify this issue, one of the co-authors of the study annotated the images used in the final analysis. Our subsequent evaluation indicates that the issue observed in the AMT results has been almost entirely resolved. Approximately 80% of the labeled images were randomly selected for training, with the remaining 20% set aside for validation. During training, the algorithm adjusts its parameters to minimize the discrepancies in patterns between predicted outputs and true labels. Upon completion of training, the algorithm was tested with the images in the validation dataset. The accuracy of the algorithm was then assessed by comparing its predictions with the true labels.

Given our focus on the algorithm’s capacity to accurately detect the objects, we chose precision, recall, and mean average precision at 50 (mAP50) scores in algorithm evaluation. We also made efforts to optimize the algorithm’s overall performance in terms of speed, efficiency, and computational complexity.

The data acquisition process was achieved through Python and the Google Street View API. The training and testing phases, based on YOLOv5, were carried out on an Nvidia GeForce RTX3080Ti GPU with 12 Gigabytes of Video-Ram. With a default learning rate and a batch size of 10, training the algorithm with a dataset comprising 3995 images took approximately one hour.

## Results

The training dataset has 3995 images, wherein a total of 9310 instances were manually labeled. That includes 4253 instances of front-wheel drivers, 1406 instances of rear-wheel passengers, and 3651 instances of helmets.

Table 1 elucidates the confusion matrices for the three object categories. As a commonly used metric for classification models, a confusion matrix illustrates the true positive (TP), true negative (TN), false positive (FP), and false negative (FN) predictions formulated by the model. Many other performance metrics can be calculated from the confusion matrix, such as accuracy, precision, recall, and the F1-score. As Table 1 indicates, the algorithm demonstrates remarkable performance in the training data. For example, the algorithm correctly detected 1127 of the 1181 front-wheel drivers.

**Table 1:** Confusion matrix

Class	TP	FP	TN	FN	Total Instances
Driver + front wheel	1127	161	N/A	54	1334
Passenger + rear wheel	227	20	N/A	32	277
Helmet	1236	30	N/A	95	1373
Total	2590	211	N/A	181	2984

Notes: TP = true positive; FP = false positive; TN = true negative; FN = false negative; The Total Instances count does not precisely equate to the sum of TP, FP, and FN due to the potential presence of multiple objects of the same class within an image

$$Precision=\frac{TP}{TP+FP}$$

$$Recall=\frac{TP}{TP+FN}$$

The validation dataset comprised 1257 images, encompassing a total of 2984 instances. That includes 1334 front-wheel drivers, 277 rear-wheel passengers, and 1373 helmets.

**Fig. 3:**
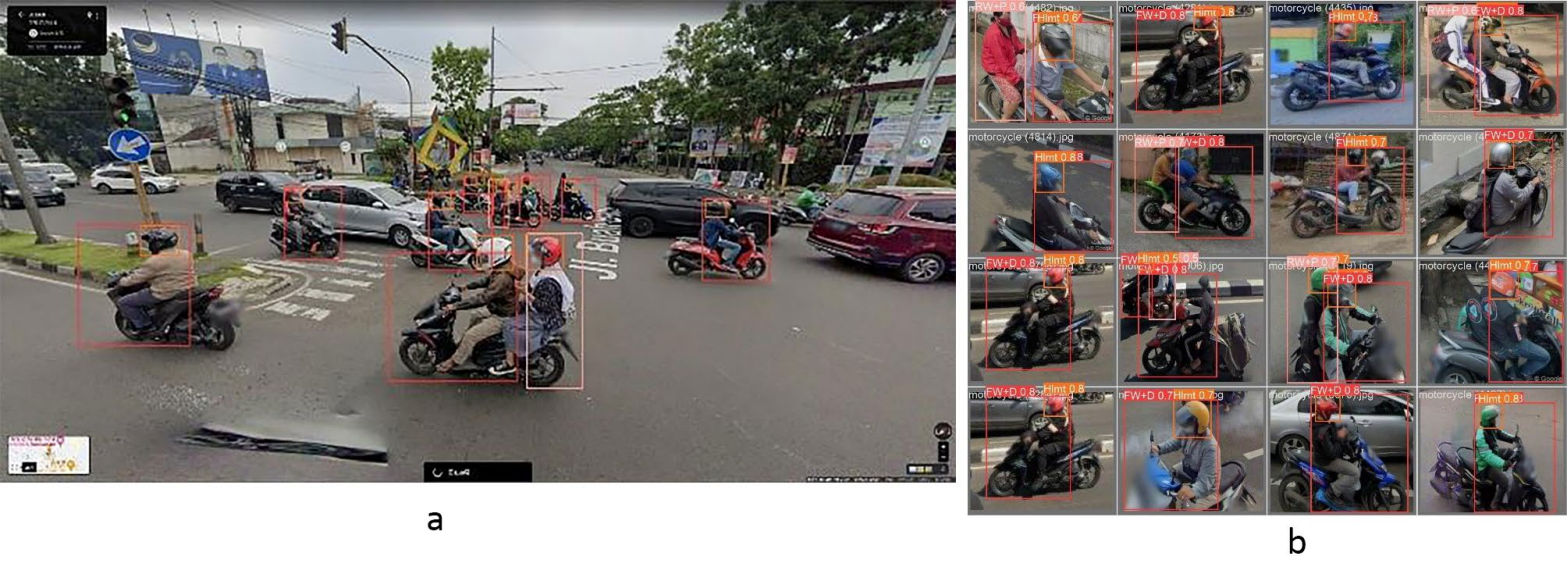
(**a**): output from the algorithm (**b**): more examples of algorithm output Examples of model performance in detecting helmets, drivers, and passengers

As illustrated in Fig. 3, the algorithm demonstrated superior performance overall. For front-wheel drivers, the algorithm achieved a precision score of 0.88, signifying that 88% of instances predicted as positive are indeed true positives (Table 2). A high precision score of this magnitude is indicative of an exceptionally low false positive rate. Concurrently, the algorithm also achieved a high recall score of 0.959, indicating that it is capable of detecting 95.9% of all true positive instances in the testing dataset.

**Table 2:** Model performance metrics in the evaluation dataset

Class	Total instances	Precision	@Recall	mAP50
Driver + front wheel	1334	0.88	0.959	0.967
Passenger + rear wheel	277	0.926	0.884	0.925
Helmet	1373	0.975	0.923	0.975
All	2984	0.927	0.922	0.956

Due to the inherent tradeoff between precision and recall, it is imperative for an object detection algorithm to strike a balance between those two scores. Therefore, our evaluation metrics also include the mean average precision value (mAP) defined below [22].

$$AP={\int }_{0}^{1}P\left(R\right)dR$$

$$mAP=\frac{1}{C}{\sum }_{i=1}^{C}AP\left(i\right)$$

where $P\left(R\right)$ denotes the accuracy $P$ corresponding to different recall rates $R$ and corresponds to the area under the P-R curve. In the $mAP$ formula, $C$ is 3, representing the three object classes detected in the study. Combining precision and recall over multiple thresholds, mAP is a more comprehensive evaluation metric. In particular, this study used the mAP50 score, which equates to how well the algorithm overlaps with a segmentation mask around at least 50% of the ground truth outline of the instance. The higher the score, the more accurate the model is at overlapping the segmentation mask. A mAP50 score of 0.967 corroborates the algorithm’s ability to detect the majority of positive instances while maintaining a low false positive rate. As such, it achieves high precision and recall scores across an array of thresholds. Comparable performance was observed in detecting rear-wheel passengers and helmets.

**Fig. 4:**
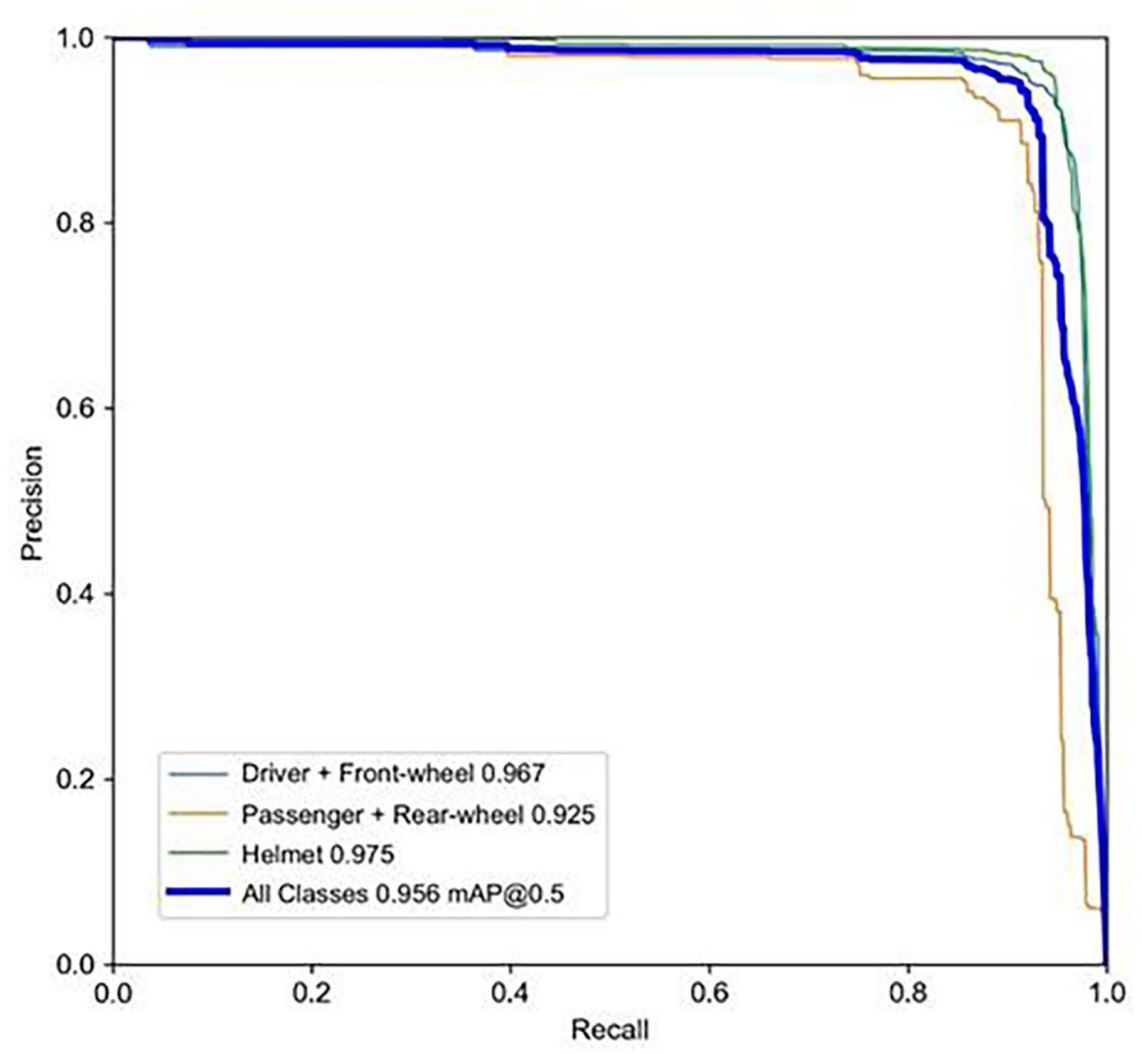
Area under the Precision-Recall Curves (AUC-PR) for the three object categories

The precision-recall curves in Fig. 4 illustrate precision and recall rates across a range of classification thresholds. Those high mAP50 scores for all object categories suggest that our algorithm performed well in identifying true positive instances while minimizing the false negative rate.

The helmet wearing rate is calculated as the number of motorcyclists wearing a helmet divided by the total number of motorcyclists. Our algorithm estimated a wearing rate of 83%, while the ground truth stands at 92%. The two figures seem close enough to render the results practically useful for real-world monitoring and evaluation purposes. As illustrated in Table 1, the primary cause of underestimation in our algorithm stems from falsely detected drivers and passengers. The algorithm misclassified other road users, such as pedestrians and bicyclists, as drivers or passengers. Although incorporating a wheel into our class definition might have mitigated this issue, further research is necessary to prevent such misclassifications.

In conclusion, the evaluation metrics lend credence to the ability of the algorithm to accurately detect motorcyclists and helmets, thereby providing a robust foundation for the estimation of helmet utilization rates.

## Discussion

The high burden of injuries and deaths among motorcyclists calls for a comprehensive understanding of the current situation and effective interventions to promote helmet-wearing. Capitalizing on the prowess of deep learning, coupled with the copious availability of Street View imagery, we introduce an algorithm endowed with the capacity to provide precise and contemporaneous information on global helmet-wearing.

Our validation results substantiate the elevated accuracy of the proposed algorithm in detecting the object categories required to estimate helmet-wearing rates. The algorithm’s remarkable performance may be attributed to YOLO and our image pre-processing. The cropping of motorcycles has proven advantageous in augmenting the algorithm’s focus on pertinent features.

Like many computer vision algorithms, identifying the precise reasons for misclassification can be challenging. Nevertheless, through manual inspection of misclassified instances, we observed that the accuracy of the algorithm was affected by the image’s perspective and the size of objects within it. By constraining the image perspectives or object sizes, the algorithm’s accuracy may be further improved.

Moreover, the common visual characteristics and injury mechanisms between cyclists and motorcyclists imply that an adapted version of the proposed algorithm could be applied to cyclists, who have been identified as another major type of vulnerable road users by the World Health Organization. That extends the applicability of the proposed algorithm to two of the three types of vulnerable road users.

Despite our extensive efforts, the study still has several limitations. Since the algorithm is trained using a labeled dataset from only one city, the algorithm may need more fine-tuning on more diverse datasets in order to improve its generalization performance. This consideration becomes crucial, particularly if the algorithm is expected to function optimally across a multitude of global regions, where the visual attributes of motorcycles and helmets, such as color, size, and shape can vary significantly. The reliance on manual labeling presents another limitation, given its inherent propensity for human error and the substantial investment of time it necessitates. To expand the applicability of the algorithm to a global level, which requires a much larger number of images, the adoption of automated image-labeling techniques may be warranted.

Our algorithm facilitates accurate estimation of helmet-wearing in any locale where Street View is accessible. The periodic updates of the Street View image collection enable the acquisition of information concerning temporal changes, particularly in major urban environments where image updates are conducted frequently. As demonstrated in Fig. 1, Street View surveys were conducted seven times in Bandung from October 2014 to August 2022. That presents an invaluable opportunity to track the changes over that period. Such temporal shifts at the municipal level could be particularly insightful if the city in question has implemented certain helmet-related policies and interventions.

Given the vast and virtually cost-free repository of imagery, our proposed algorithm lends itself readily to integration into both national and international monitoring frameworks. The regularity of Street View updates implies that recent data can be leveraged by governmental authorities to oversee compliance with helmet-wearing regulations and to allocate resources efficiently.

The geospatial feature of the results generated by our algorithm empowers urban planners and traffic engineers to pinpoint high-risk areas where helmet use is suboptimal. Such information can inform the creation of targeted interventions, such as educational campaigns [23]. 

The standardization of our algorithm with respect to deep learning and data sourcing renders it eminently suitable for international benchmarking and the tracking of progress. The generated estimates are highly comparable across different countries, thereby facilitating the identification of best practices and coordination of efforts to enhance helmet-wearing globally. For instance, Target 7 of WHO’s Global Road Safety Performance Targets aims to increase the proportion of motorcycle riders correctly using standard helmets to close to 100% by 2030.

The Google Street View API is a paid service, but new users receive a free credit along with a monthly renewed allowance. This allocation should enable the acquisition of tens of thousands of images each month, which is typically adequate for the proposed algorithm’s primary use case: providing decision-makers with estimates for their city or region.

However, the API cost may pose a barrier for large-scale implementations, such as global situation assessments. Considering the similarities between Google Street View and alternative no-cost street view data sources, we believe that the algorithm can be readily applied to images obtained from alternative sources, such as Mapillary.

To conclude, our proposed algorithm affords local, national, and international stakeholders an accurate and up-to-date understanding of helmet-wearing behaviors. That allows for the development of targeted interventions and the tracking of progress in the ongoing efforts to reduce road traffic injuries and fatalities.

## Data Availability

The datasets generated and/or analyzed during the current study are publicly available at https://github.com/QFL2020/helmet.
